# Colorimetric Phosphate Detection Using Organic DFB Laser Based Absorption Spectroscopy

**DOI:** 10.3390/mi12121492

**Published:** 2021-11-30

**Authors:** Thilo Pudleiner, Elias Sutter, Jörg Knyrim, Christian Karnutsch

**Affiliations:** Institute for Sensor and Information Systems, Research Group Integrated Optofluidics and Nanophotonics (IONAS), University of Applied Sciences Karlsruhe, 76133 Karlsruhe, Germany; esutter@outlook.de (E.S.); Joerg.Knyrim@h-ka.de (J.K.)

**Keywords:** organic laser, DFB laser, optofluidics, polymer laser, absorption spectroscopy, phosphate, bilateral emission, photometer, laser absorption spectrometer

## Abstract

A novel compact laser absorption spectrometer is developed for colorimetric detection. We demonstrate the realization of the system as well as example measurements of phosphate in water samples based on the malachite green (MG) method. A phosphate concentration range of 1 mg/L to 31.25 μg/L (which corresponds to a molar concentration range of 10.5 μmol/L to 329 nmol/L) is investigated. This photometer demonstrates the ease of integration of organic distributed feedback (DFB) lasers and their miniaturizability, leading the way toward optofluidic on-chip absorption spectrometers. We constructed an optically pumped organic second-order DFB laser on a transparent substrate, including a transparent encapsulation layer, to have access to both emission directions of the surface-emitting laser. Using the two different surface emission directions of the laser resonator allows monitoring of the emitted light intensity without using additional optical elements. Based on these advances, it is possible to miniaturize the measurement setup of a laser absorption spectrometer and to measure analytes, such as phosphate.

## 1. Introduction

Phosphate is one of the most common plant and animal nutrients. Phosphate is ubiquitous in the environment and is subject to a natural cycle, the balance of which has a strong impact on animal and plant life [1]. The management of phosphate is critical to maintaining desirable water quality and ecosystem integrity. Thus, excess phosphate mainly contributes to the eutrophication of surface waters, such as lakes, rivers, reservoirs or streams [2,3]. Eutrophication is the excessive enrichment of water bodies with mineral nutrients and leads to the explosive growth of autotrophic organisms, such as algae and cyanobacteria [1], which causes mass mortality and reduced biodiversity in these environments due to the high oxygen demand. Caused by the explosive growth, eutrophication is one of the most visible types of man-made pollution of surface waters. In addition to fertilizer use, untreated industrial wastewater, agricultural wastewater and animal waste are additional sources of phosphates and other nutrients that disrupt the natural phosphate cycle [1,2,4]. To avoid this disturbance of flora and fauna, it is important to monitor wastewater to detect excess nutrients and react in time. This requires measurement methods that are simple and accurately detect low levels of excess nutrients, for example, orthophosphate. Orthophosphate is an anion of orthophosphoric acid and belongs to the group of phosphates. Therefore, orthophosphate is the only form of phosphorus that autotrophs can assimilate [2]. Therefore, the greatest attention is paid to the detection of orthophosphate. All measurements in this work detect orthophosphate, which is referred to as phosphate in the following.

Samples are often collected manually and analyzed in laboratories. The need for laboratories is time consuming and expensive [5]. Furthermore, this often results in a loss of information about the time course of nutrient overloads. Field measurements, in contrast, which allow for operational monitoring of wastewater, can make it possible to protect water systems from overloading. It is also possible to localize polluters in a system.

There are several possible solutions for such sensors or sensor network applications. The most promising are electrochemical and spectrophotometric sensors. Electrochemical sensors are highly suitable for miniaturization and can therefore be manufactured inexpensively, and the energy requirement can also be reduced. However, electrochemical sensors often suffer from low selectivity or low sensitivity [6,7,8]. Spectrophotometric methods usually require complex, expensive and bulky equipment and are typically not amenable to field measurements [6]. Spectrophotometric sensors use color-forming reactions and thus, generate special waste. This waste has to be collected and can become a problem, especially in small, stand-alone systems [8,9]. Spectroscopic detection is a photometric measurement of absorption at a small spectral bandwidth. Photometry makes it possible to work very selectively and sensitively with a high stability. Therefore, the adaptation to field applications of such systems has a particularly high priority.

The limited usable spectral bandwidth due to color-forming reactions restricts the miniaturization of a sensor in several ways. This includes spectral limiting of the emission source. Commercial spectrometers often use broadband light sources in combination with monochromators. The monochromators usually consist of movable optics or gratings, which allow a separation across the spectrum, but also make such systems expensive and bulky.

A possible remedy is the use of an emission source with a narrow spectral emission bandwidth. This is often achieved by the integration of inorganic emission sources, such as light-emitting diodes or laser diodes, which have a limited emission bandwidth due to their discrete optical transitions [8,10]. Furthermore, organic emission sources are also used [11,12], which can cover the entire visible spectrum (and beyond) [12,13,14]. Therefore, organic emission sources can be used to detect many different color-forming reactions.

The reduction in the available optical power by using a small emission bandwidth is another limitation. The use of an organic laser shall overcome both challenges: a typical narrow bandwidth for a selective measurement in combination with a high optical power achieved by laser oscillation.

In this paper, we demonstrate a novel spectrometric phosphate detection unit based on organic DFB lasers using the MG method. The design and fabrication of the laser cell are described, and measurements in a phosphate concentration range of 1 mg/L to 31.25 μg/L are presented.

## 2. Experimental

The measurement of the absorbance of a colorimetric reaction is tied to the sample geometry. The absorbance is exponentially coupled to the optically penetrated distance. For this purpose, a laser is set up and its emission is attenuated when it passes through a phosphate-selective color-forming reagent.

### 2.1. Design and Fabrication

In our work, the emission source is an organic second-order DFB laser in a sandwich construction. Optical emission is achieved through a thin layer of an organic semiconductor polymer. Organic semiconductors are conjugated molecules, with the semiconducting properties arising from the overlap of molecular orbitals [14]. Organic semiconductors, which intrinsically offer a four-level laser architecture, achieve their gain by the recombination of excited singlet exciton states. While excited singlet states contribute to optical gain, other excited states, such as triplet excitons and polarons, are considered detrimental to lasing [15]. Both triplet excitons and polarons are the major product of charge recombination [15,16]; thus, efficient organic lasers mostly employ pulsed optical pumping [17,18] (see section Optical Setup below). In addition to the pump configuration used in this work, it is also possible to pump the organic laser with a pulsed inorganic light emitting diode or laser diode [19,20,21].

The organic semiconductor materials used have high absorption for the pump radiation [16], which enables a thin nanometer-scale organic layer to act as a planar waveguide. The superposition of frequency-selective diffraction at a spatial corrugation of the boundary surface and the guiding planar layer leads to a DFB resonator structure.

**Figure 1:**
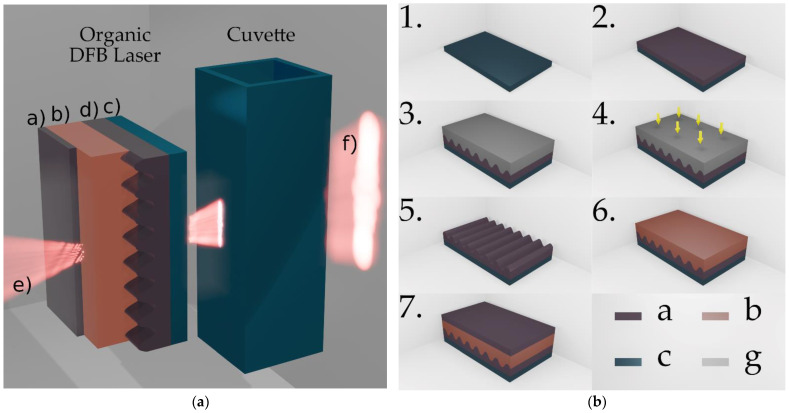
Laser design: (**a**) Three-dimensional illustration of a surface-emitting DFB laser with a standard glass cuvette transmitted by the bottom emission of the laser (not to scale): (a) encapsulation layer; (b) active organic layer and core of the planar waveguide; (c) auxiliary substrate (microscope glass slide); (d) DFB resonator substrate; (e) top emission; (f) bottom emission (**b**) illustration of the fabrication process steps of the organic second-order DFB laser (not to scale); 1. Auxiliary substrate; 2. Apply MD700 film; 3. Positioning of master on MD700; 4. UV-exposure through master; 5. Removing master; 6. Apply active organic emission layer; 7. Encapsulate with MD700 (Materials: a, MD700; b, conjugated polymer; c, glass; g, fused silica.

The process steps for manufacturing the organic DFB laser are illustrated in Figure 1b. A schematic cross section of the device is shown in Figure 1a. The three functional laser layers DFB substrate, organic emission layer and encapsulation layer are stacked on a commercially available microscope glass slide, which from now on is denoted as an auxiliary substrate (see Figure 1b Step 1).

The DFB substrate carries a sinusoidal one-dimensional grating with a period of Λ=400 nm. In order to create the DFB substrate, a replica of a master grating was molded into a Fluorolink^®^ MD700 (PFPE-urethane methacrylate) layer on top of an auxiliary substrate. Here, the master grating used is a segment of a patterned fused silica wafer produced by laser interference lithography. MD700, diluted with 2% by weight of the photoinitiator Darocur^®^ 1173, was deposited on the auxiliary substrate (Figure 1b Step 2). The master was lightly pressed into the MD700 with the patterned surface pointing downwards (Figure 1b Step 3). The ultraviolet (UV)-curable MD700 was exposed for 60 s through the master using a mercury vapor lamp with a power density of $800\;\mathrm{mW}\cdot {\mathrm{cm}}^{-2}$ (Figure 1b Step 4). Subsequently, the master can be removed and later be reused, the crosslinked MD700 now carries a negative replication of the master grating (Figure 1b Step 5). The depth of the sinusoidal structure in the MD700 with $d_{st}=38.16\;\mathrm{nm}\pm 0.37\;\mathrm{nm}$ agrees well with the structure depth of the glass master with $d_{st}=38.74\;\mathrm{nm}\pm 0.62\;\mathrm{nm}$. The active organic emission layer consists of a guest–host system of MEH–PPV (ADS100RE) and F8BT (ADS233YE). The guest, poly(2-methoxy,5-(2′-(ethyl)hexyloxy)-p-phenylene vinylene), and the host, poly(9,9-dioctylfluorene-alt-benzothiadiazole), were dissolved in toluene at an experimentally optimized weight ratio of 6%:94%. In a spin coating process, the active organic material was applied to the DFB substrate at a spin speed of 750 rpm in a toluene-saturated atmosphere (Figure 1b Step 6). This results in an approximately 320 nm thick layer that forms the optical waveguide of the laser cavity. To reduce photooxidation and increase the life expectancy of the laser, the organic layer is encapsulated with an additional MD700 layer (Figure 1b Step 7). As illustrated by the beam lobes in Figure 1a, all layers have a high transmittance in the visible spectrum and laser emission can be radiated in bottom and top directions.

### 2.2. Methods and Materials

The basic detection principle follows the Beer–Lambert law, a traditional theory of attenuation of radiation intensity when passing an absorbing sample. In our case, attenuation of transmitted light is used to determine an analyte concentration. This can be expressed as follows:

(1) $$A_{\lambda }=log\left(\frac{I_{T}}{I_{0}}\right)=\epsilon_{\lambda }\cdot c\cdot d$$

where $A_{\lambda }$ is the absorbance of the sample at a specified wavelength λ, $I_{0}$ is the initial light intensity before passing the sample, $I_{T}$ is the transmitted light intensity behind the sample, d is the optical path length, $\epsilon_{\lambda }$ is the specific extinction coefficient of the analyte, and c is the concentration of the analyte. For the determination of the absorbance according to Beer–Lambert, the initial light intensity and the transmitted light intensity behind the sample, independent of other losses, must be known.

**Figure 2:**
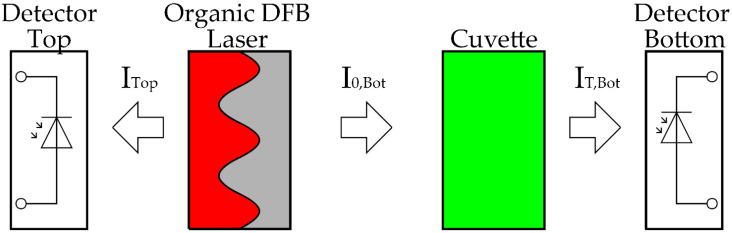
Schematic layout of the emitted radiation of the organic DFB laser with cuvette.

A beam splitter is conventionally used to determine the initial intensity $I_{0}$. Using a beam splitter increases the complexity of miniaturized setups, which is why the excitation energy of the emission source is often used as a measure of the emission intensity [8,22]. This approach simplifies the measurement setup but runs the risk that changes in the emission source intensity will lead to measurement errors. To avoid both problems, we used the surface emission in two different directions of an organic second-order DFB laser [23]. A DFB resonator grating diffracts an incident wave into transmitted and reflected orders, comparable to a beam splitter. As shown in Figure 2, the emission intensity of the organic DFB laser is split into top $\left(I_{Top}\right)$ and bottom $\left(I_{0,Bot}\right)$ directions. The two surface emissions are based on the same diffraction condition and are identical in emission wavelength and emission intensity due to the symmetry of the layers [24].

To use the two-sided emission for absorbance measurements, the laser is placed between two detectors. One detector is arranged for each surface emission in top and bottom emission directions. This provides the possibility to measure intensity in the top and bottom directions for each laser pulse. The fluid to be measured is placed between the laser and a detector. The cuvette containing a sample is therefore in the beam path of the bottom emission of the laser (see Figure 2). As mentioned above, the absorbance can be determined with the help of the Beer-Lambert law. The optical attenuation considered here is only due to the absorption of an optical wave by a medium. To determine the absorbance, at least two measurements must be made.

A reference measurement is performed, where the laser emission transmits a cuvette filled with deionized (DI) water, and a solution measurement is performed, where the emission transmits a cuvette filled with a measurement solution. Each of the two measurements generates two values: the intensity in the top direction $\left(I_{Top}\right)$ and the intensity in the bottom direction $\left(I_{T,Bot}\right)$. Both intensities are then used to calculate a reference ratio $\left(R_{Reference}\right)$ and a solution ratio $\left(R_{Solution}\right)$ (Equations (2) and (3)).

(2) RReference=(IT,BotITop)Reference=I0,BotITop

(3) RSolution=(IT,BotITop)Solution=IT,BotITop

(4) RSolutionRReference=IT,BotI0,Bot=ITI0

(5) Aλ=log(ITI0)=log(IT,BotITopI0,BotITop)

The two emission ratios differ in scattering and absorption losses during optical penetration of the cuvettes. The reference ratio $\left(R_{Reference}\right)$ includes losses due to scattering as well as absorption caused by the DI water and the cuvette. The solution ratio $\left(R_{Solution}\right)$ includes approximately the same scattering losses as the reference ratio. In addition, the solution ratio also depends on the absorption losses of the analyte. When additional scattering in the measurement solution is negligible and the emission ratio of the laser is constant in the top and bottom directions, the ratio of $\left(R_{Solution}\right)$ and $\left(R_{Reference}\right)$ is equal to the ratio of the intensity before and after passing the absorbing solution (Equation (4)). Hence, Equation (5) shows the Beer–Lambert law (Equation (1)) with substituted intensity ratios.

Here, the analyte to be detected is phosphate. Since phosphate itself has no absorbance line in the visible spectrum, a color forming reaction is used for detection [5,10,25,26]. To determine the phosphate concentration, a colorimetric reaction known as the MG method is used. It has a fast color forming reaction without heating, and a larger sensitivity than the mostly used molybdenum blue reaction [26].

### 2.3. Chemicals and Reagents

For phosphate detection, a working solution of the analyte (phosphate) and a MG reagent are mixed. For all stock and calibration solutions, DI water produced by an ultrapure Direct-Q Water treatment system was used, which has a conductivity of 0.04–0.05 μS/cm.

The MG reagent is based on three stock solutions. A 24% sulfuric acid was prepared from a commercially purchased solution of 25% sulfuric acid ($H_{2}{\mathrm{SO}}_{4}$; Merck Chemicals Ltd., Darmstadt, Germany). Then a 0.1 mol sodium molybdate solution was prepared by mixing 500 mL DI water and 10 g sodium molybdate dihydrate (${\mathrm{Na}}_{2}{\mathrm{MoO}}_{4}\cdot 2H_{2}O$; Merck Chemicals Ltd., Darmstadt, Germany).

To obtain a homogeneous fluid phase [5,25], a protective colloid based on 10 g poly(vinyl alcohol) (PVA) (${\left[CH_{2}CH\left(OH\right)-\right]}_{n}$; Merck Chemicals Ltd., Darmstadt, Germany) was dissolved in 1 L DI water heated to near boiling point. This solution was filtered with a folded filter paper disc (Grade 589/3 blue) and poured into a graduated flask. Then, 100 mg of MG G ($C_{27}H_{34}N_{2}O_{4}S$; Thermo Fisher GmbH, Kandel, Germany) was added to 270 mL of the PVA solution. This solution was homogenized and mixed with DI water to provide a total volume of 500 mL of the MG reagent.

On the basis of a potassium phosphate stock solution ($KH_{2}PO_{4}$; Merck Chemicals Ltd., Darmstadt, Germany) with a phosphate concentration of 1000 mg/L, different phosphate working solutions were prepared. The used phosphate working solutions correspond to a phosphate concentration range from 2 mg/L to 31.25 μg/L. To achieve a high accuracy of the phosphate working solutions, only the highest phosphate working solution (2 mg/L) was prepared from the 1000 mg/L phosphate solution. The highest working solution (2 mg/L) was then diluted to the smaller phosphate working solutions of 1 mg/L, 500 μg/L, 250 μg/L, 125 μg/L, 62.5 μg/L and 31.25 μg/L by mixing with DI water. In addition, a 375 μg/L phosphate working solution was prepared from the 0.5 mg/L phosphate working solution.

### 2.4. Measurement Procedure

Phosphate detection based on the MG method enables the detection of low concentrations and provides low cross sensitivity [25,27]. For our measurements, the above-mentioned stock solutions were mixed, which resulted in a MG reagent. This MG reagent together with the phosphate sample led to the phosphate detection. The MG G solution (0.42 mL) was added to 24% sulfuric acid (0.25 mL) and filled with 0.1 mol sodium molybdate solution (0.42 mL). After 10 min color development of the MG reagent (1.09 mL), a phosphate sample (2.08 mL) was pipetted in. The MG reagent together with a phosphate working solution was filled into a cuvette, denoted from now on as “MG+P”. The cuvette was left to rest for 60 min for colorimetric development before measuring the absorbance.

The absorbance of every sample was measured in the organic DFB laser absorption spectrometer (see Figure 1a and Figure 2) and, for comparison, it was also measured using a commercial UV-VIS spectrometer (UV-1800; Shimadzu, Kyōto, Japan). The detection range of the MG method lies between 10 μg/L and 1.2 mg/L phosphate [25]. At higher phosphate concentrations of more than 2.5 mg/L, turbidity occurs in the sample, which makes photometric absorption measurements impossible [10].

### 2.5. Optical Setup

**Figure 3:**
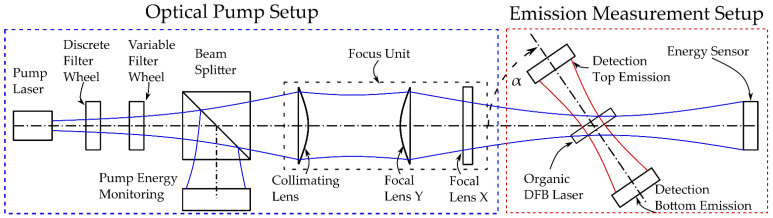
Schematic of the optical pump setup (with focus unit) and the emission measurement setup of an organic DFB laser with two different measurement channels (top emission and bottom emission).

**Figure 4:**
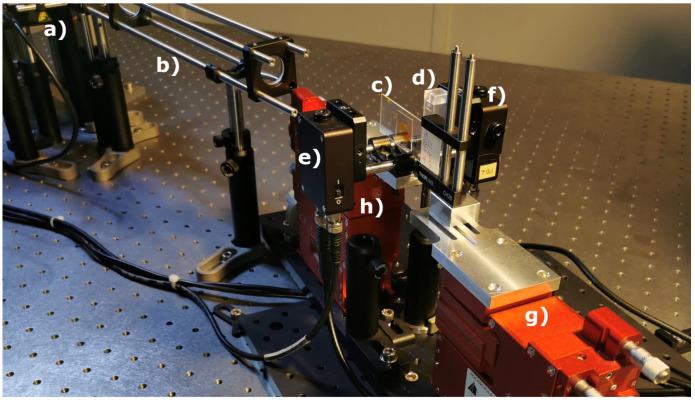
Image of the optical emission measurement setup; (**a**) pump laser, filter and monitoring, (**b**) focus unit, (**c**) organic DFB laser, (**d**) cuvette, (**e**) photodetector top emission, (**f**) photodetector bottom emission, (**g**) translation stage organic DFB laser, (**h**) translation stage laser detection unit.

Figure 3 and Figure 4 outline the optical pump setup and emission measurement setup of the organic DFB laser in our laboratory. With a cuvette being placed between the organic laser and the bottom emission detection (Figure 4d)), we also used this setup as our organic laser absorption spectrometer. Sketched in blue is the UV pump radiation required to optically pump the organic DFB laser. The pump pulse of a frequency tripled passively Q-switched ND:YAG laser (FTSS355-Q2; CryLas, Berlin, Germany) with a wavelength of $\lambda_{pump}=355\;\mathrm{nm}$ exhibits a pulse duration of 1.9 ns. All measurements are carried out at a pump pulse repetition rate of 1 Hz. The use of neutral density (ND) filters as a variable ND filter mounted on a stage combined with a revolver equipped with discrete ND filters allows variation of the pump energy. A beam splitter (92:8) was positioned in the pump beam behind the ND filters for pump pulse energy monitoring. Pump energy calibration of the monitor photodiode was performed using an energy sensor (Pe10b; Gentec, Quebec, QC, Canada). A focusing unit consisting of a collimator and two plano-convex lenses was used to create an elliptical pump spot with variable beam diameter in length ($\omega_{y}=180\;\mu m$ to 1500 μm) and fixed diameter in width ($\omega_{x}=150\;\mu m$). The organic DFB laser was arranged at an angle ($\alpha =82^{{}^{\circ}}$) in the pump beam. The oblique pump configuration at a steep angle allows the measurement of organic laser emission at small distances without affecting the pump radiation. In addition, the steep angle prevents the sample fluid from being exposed to the UV pump source. The actual pump spot on the surface of the organic laser thus also exhibited an elliptical shape but with transformed dimensions ($\omega_{y}=1497\;\mu m$, $\omega_{x}=1078\;\mu m$), with the longer beam waist oriented orthogonally to the grating lines of the DFB resonator. The organic DFB laser emission in both directions is outlined in red in Figure 3. The detection of the emission of the organic DFB laser as well as the monitoring of the pump emission was done with amplified Si Photodetectors (PDA10A; fixed gain; Thorlabs, Newton, MA, USA). In order to calibrate the pump energy monitor diode, the organic DFB laser was removed from the setup and an energy sensor was used instead. The photodetectors were read out with an oscilloscope (MSO9254A; Agilent, Santa Clara, CA, USA). To avoid discrepancies in bottom and top emission detection, nominally identical photodiodes and connectors were used.

As mentioned above, we used an organic DFB laser as emission source for absorption spectroscopy. A DFB resonator generates feedback based on a periodically distributed change of the refractive index and the optical gain. Resonance for the guided mode is enabled in close vicinity of the Bragg wavelength ($\lambda_{Bragg}$) given by the Bragg condition [14,28].

(6) $$\Lambda =\frac{\lambda_{Bragg}\cdot N}{2\cdot n_{eff}\left(\lambda \right)}$$

where Λ is the period of the spatial modulation, $n_{eff}\left(\lambda \right)$ is the effective refractive index of the guided mode, and N is the order of diffraction. Thus, the Bragg condition describes the wavelength-selective resonance based on the diffraction of a guided mode at a periodically changing interface [29]. A change in lasing wavelength can be achieved by changes in periodicity [21,30,31] or guidance of the mode. One common way to change the guiding of the mode is to vary the thickness of the core layer of the planar waveguide [14,32,33]. The laser emission wavelength can thus be varied through changing the thickness of the organic layer that forms the core layer of the waveguide.

**Figure 5:**
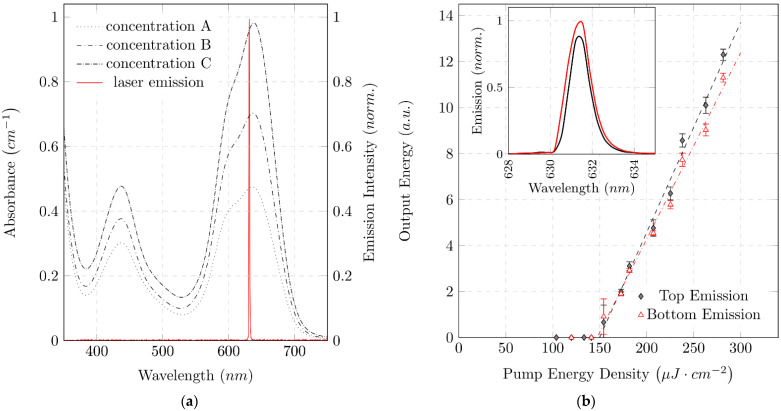
Laser emission characteristics and spectral absorption of colorimetric reaction: (**a**) Absorbance spectra of MG+P for different phosphate concentrations measured with a UV-VIS spectrometer. Concentrations: A=0.5 mg/L; B=1 mg/L; C=2 mg/L. Additionally, a normalized organic laser emission spectrum is shown. The emission wavelength of the laser defines the measurement wavelength and is thus optimized for a local absorption maximum of MG+P. Absorbance of MG+P shows a significant dependence on the phosphate concentration in the vicinity of the measuring wavelength; (**b**) Emission characteristics of a fabricated organic DFB laser for both top and bottom emission: optical output pulse energy versus optical pump energy density and emission spectra (inset). Almost identical laser threshold energy density of about $150\;\mu J\cdot {\mathrm{cm}}^{-2}$ in top and bottom emission direction is observed.

Variation of the emission wavelength offers the possibility to optimize the laser to the color forming reaction for measuring a specific analyte. Figure 5a plots the absorbance of MG+P in the visible spectrum for three different phosphate concentrations. In the visible spectrum, MG+P shows two dominant absorption peaks. The two peaks $\lambda_{1}=439\;\mathrm{nm}$ and $\lambda_{2}=634\;\mathrm{nm}$, mark possible measurement wavelengths. Both absorption peaks could potentially be used to determine the phosphate concentration. Our absorbance measurements were performed at $\lambda_{2}=634\;\mathrm{nm}$ because this peak offers the highest measurement sensitivity. Our organic laser was designed for an emission wavelength close to the absorption maximum $\lambda_{2}=634\;\mathrm{nm}$. The resulting emission spectrum of the organic laser with a peak wavelength of $\lambda_{Las}=631\;\mathrm{nm}$ is shown in Figure 5a.

Optical alignment is performed with two five-axis translation stages (i5000; Luminos, Ottawa, Canada). The organic DFB laser is mounted on one stage (Figure 4g) and the detection instruments for recording the top and bottom organic laser emission on another (Figure 4h). In order to record both the optical spectrum and the intensity, the detection unit is mounted in a cage system (SR 30mm; Thorlabs, Newton, USA) on the translation stage. The fibers of the fiber-coupled spectrometer can easily be replaced by photodiodes without change in alignment. Furthermore, the detection units for top and bottom emission are mechanically connected to each other and can be moved symmetrically around the organic DFB laser. The emission spectrum was used as orientation for the alignment of the optical measuring equipment. The shift of spectral emission as a function of divergence angle of the organic DFB laser must be taken into account when aligning the measuring equipment [34]. Using two multimode fibers (d=600 μm) and a spectrometer, the fibers fixed to each other are aligned to the pump spot. Using rotational alignment of the fibers around the pump spot, fine tuning in the adjustment of the spectral emission is achieved. The inset in Figure 5b shows emission spectra in top and bottom direction, measured with a spectrometer (USB 2000; Ocean Optics, Orlando, FL, USA). The emission spectra in the different emission directions show hardly any spectral differences. The peak wavelengths are $\lambda_{Bottom}=631.42\;\mathrm{nm}$ and $\lambda_{Top}=631.38\;\mathrm{nm}$, and the spectral bandwidths are $\Delta \lambda_{\mathrm{FWHM}-\mathrm{Bottom}}=1.325\;\mathrm{nm}$ and $\Delta \lambda_{\mathrm{FWHM}-\mathrm{Top}}=1.115\;\mathrm{nm}$. The emission in the top direction is approximately 0.83 times smaller than the bottom emission. To avoid spectral differences due to measurement set up, both spectra were measured with the same spectrometer. Therefore, the spectrum of the pulsed laser was measured in each emission direction one after the other. The emissions in top and bottom direction are, thus, not associated with the same pump pulse. Any deviation in the pump pulse causes deviations in emission intensity. Both measurement fibers showed no discernible deviation in alignment to the surface normal of the organic laser. Fibers were positioned at a distance of 25 mm to the laser.

Examination of the laser characteristics is a common option to prove laser activity [35]. Thus, the optical output energy is plotted in Figure 5b for different pump energies. Laser action occurs above the laser threshold, where the slope efficiency is dramatically increased by the transition from spontaneous emission to stimulated emission. The threshold is reached at the same pump energy density for top ($D_{Top}=149.90\;\mu J\cdot {\mathrm{cm}}^{-2}\pm 12.3\;\mu J\cdot {\mathrm{cm}}^{-2}$) and bottom ($D_{Bottom}=147.14\;\mu J\cdot {\mathrm{cm}}^{-2}\pm 15.6\;\mu J\cdot {\mathrm{cm}}^{-2}$) emissions. The emission ratio of the organic DFB laser in top and bottom emissions appears to differ during measurement of the laser characteristics (laser output energy as a function of pump energy). The ratio of the slope efficiencies of top and bottom emissions is 0.88 ± 0.035, which deviates slightly from the theoretical prediction of 1 [24]. In order to achieve different pump energy densities, the optical power of the pump pulse must be varied. This energy variation is done by mechanical ND filters. It is assumed that small mechanical interventions in the pump beam guidance, which occur by mechanically shifting the ND filters, lead to a local change of the pump spot. The local change in pump position on the organic laser could lead to a discrepancy in measurement acquisition. The photodiodes aligned in advance with the pump position then no longer measure in the center of the organic DFB laser beam. Measurements carried out after realigning the diodes gave an emission ratio of 1, as predicted by Streifer et al. [24].

### 2.6. Data Acquisition and Processing

As mentioned previously, the emission intensity is measured with photodiodes. The acquisition of the pulsed DFB laser signals with free beam photodiodes was performed at a distance of 40 mm, which compensates for the difference between the active area of the photodiode and the area of the fiber core. It is known from literature that the pulse duration of organic laser emission correlates closely with the pump pulse duration, which here is 1.9 ns [36].

**Figure 6:**
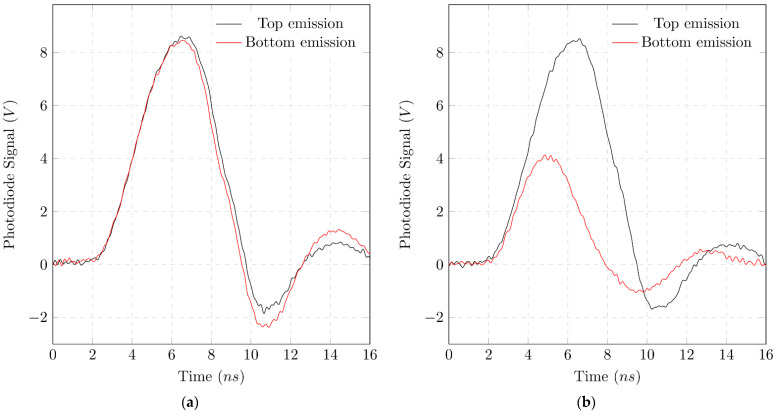
Single shot measurements of the emitted organic laser pulses in top and bottom direction. Observed pulse duration is three to five times longer than the pump pulse and varies in duration. (**a**) with a cuvette filled with DI water in the optical path of the bottom emission. This serves as a reference measurement; (**b**) with a cuvette filled with a highly absorbing sample (MG+P with 0.5 mg/L phosphate) in the optical path of the bottom emission.

Figure 6a,b show the photovoltage $U_{Diode}$ of the photodiodes over time for an organic laser pulse. The observed pulse duration is three to five times longer than the pump pulse and varies in duration.

(7) $$I=\frac{R_{L}+R_{S}}{ℛ\left(\lambda \right)\cdot G_{TI}\cdot R_{L}\cdot \Omega }\cdot U_{Diode}=\kappa_{TI}\cdot U_{Diode}$$

To determine the absorbance of a sample according to the Beer–Lambert law, the optical intensity I has to be measured (see Equation (1)). According to Equation (7), the intensity should be directly proportional to $U_{Diode}$. Our pulsed measurements suffer from low bandwidth (BW=150 MHz) of the employed photodiodes and transimpedance amplifiers. Transimpedance circuits can be described by a second-order transfer function, which leads to overshoots with a damped decaying behavior and increased rise times. Since we acquired our absorbance data by calculating signal ratios, differences in the transfer function of the measuring channels have an effect on the measured ratio. However, the use of nominally identical photodiodes, amplifiers, connectors as well as an identical data acquisition scheme keep these differences negligible. Because both laser pulses (emitted from top and bottom) originate from the same emission source, their actual pulse duration must be identical. The deformation of the signal due to the electronic circuit and its energy buffering properties can be neglected since the physical pulses differ only in their pulse height and not in their duration.

Based on this, it is possible to use the emitted pulse energy Q instead of intensity for further calculations. According to Equation (8), the energy Q of a pulse is proportional to the intensity I integrated over the pulse duration. The solid angle Ω is defined by the distances and the active area of the photodiode.

(8) $$Q=\Omega \cdot \overset{t_{1}}{\underset{t_{0}}{\int }}I\;dt$$

The emitted pulse energy Q was thus obtained by integration of the main peak of the photovoltage $U_{Diode}$.

For the determination of the absorbance, the ratio of top and bottom emission intensities is needed (see Equation (5)). As outlined in Equation (9) and explained above, $I_{Top}$ and $I_{Bot}$ can be replaced by $Q_{Top}$ and $Q_{Bot}$.

(9) Aλ=log((IT,BotITop)Reference(IT,BotITop)Solution)=log((QT,BotQTop)Reference(QT,BotQTop)Solution)=log([κTI,Bot⋅∫UDiode,BotdtκTI,Top⋅∫UDiode,Topdt]Reference[κTI,Bot⋅∫UDiode,BotdtκTI,Top⋅∫UDiode,Topdt]Solution)

## 3. Results and Discussion

**Figure 7:**
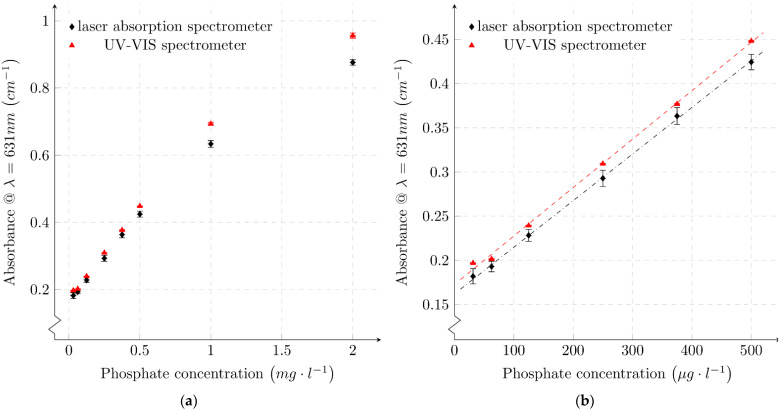
Absorbance of various phosphate samples measured with a commercial UV-VIS spectrometer and our laser absorption spectrometer: (**a**) full sample range; (**b**) linear range below 500 μg/L phosphate. Dashed lines represent the linear fit for each measurement technique.

Our absorbance measurements are presented in Figure 7. Every sample was measured in the laser absorption spectrometer and, for comparison, was also measured using a commercial UV-VIS spectrometer. For both spectrometers, measurements were made at a peak wavelength of λ=631 nm. Figure 7a shows absorbance values measured for a large dynamic range from 2 mg/L to 31.25 μg/L phosphate concentration. In Figure 7b, a partial range with small phosphate concentrations below 0.5 mg/L is plotted. The error bars represent the standard deviation determined from seven measurement points for the UV-VIS spectrometer and nine measurement points for the laser absorption spectrometer. In addition to the measurement inaccuracy, an additional error occurs due to the time-dependency of the MG phosphate color development reaction [10,37]. Considering the fact that the two systems are different in numerous aspects, the measured data agree remarkably well.

The correlation between absorbance and phosphate concentration is not linear in the colorimetric MG reaction. Absorbance measurements for concentrations above ∼0.5 mg/L indicate smaller values than expected in a linear fit, which is evident in Figure 7a. Confining the correlation between absorbance and phosphate concentration to a linear range, as shown in Figure 7b, is a common approach [10]. A linear regression of the results of the two measurement systems was calculated using the least squares method. For the determination of a linear fit of the absorbance as a function of phosphate concentration according to Equation (10), only concentrations less than 0.5 mg/L were considered. The linear fits are plotted in Figure 7b and the determined function parameters slope, offset, errors and $R^{2}$ are given in Table 1.

(10) $$A_{\lambda }=Slope\cdot c+Offset$$

**Figure 8:**
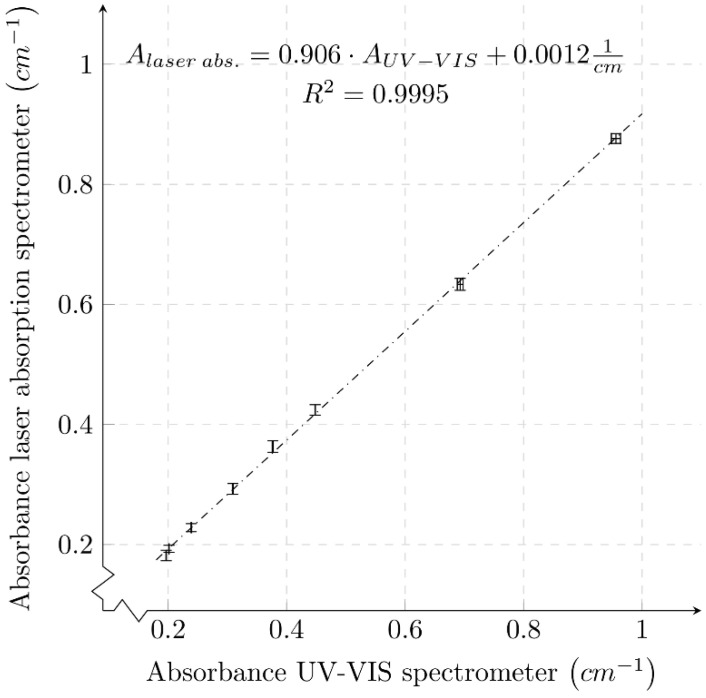
Comparison of the results of the two different measurement techniques at a measurement wavelength λ=631 nm. The slope of 0.906 of the regression line differs slightly from the expected slope of 1 for identical systems.

Figure 8 compares the absorbance derived from the two measuring systems for all phosphate concentrations from 2 mg/L to 31.25 μg/L. In addition, the standard deviations of the individual absorbance values for the UV-VIS spectrometer and the organic laser absorption measurement system are plotted. A regression line was calculated, plotted in Figure 8, which compares the behavior of the two measurement systems. The absolute values of the absorbance determined by the UV-VIS spectrometer are larger than the absorbance values determined by the organic laser absorption spectrometer for each phosphate concentration. The difference in absorbance value increases with increasing phosphate concentration. Comparing the linear fits of the results of the two measurement methods (see Table 1), both the offset and slope are lower for the laser absorption spectrometer. Taking into account the determined errors of the function parameters, a deviation beyond the error limits can be assumed only for the offset. The deviation of the slope of the laser absorption regression line is larger than that of the UV-VIS spectrometer. The larger deviation of the measurements is due to mechanical variations. The measurement routine for the laser absorption spectrometer involves replacing the cuvette each time for each measurement value. Due to variations in the position of the cuvette, the reflection and optical path are slightly changed. In the UV-VIS spectrometer, the cuvette remains in the same position for several measurements in succession; therefore, the deviation of these measurements is smaller.

The colorimetric sample for each concentration was taken from the same mixture for both measurement techniques. Furthermore, after the measurements, the samples were subjected to a control measurement with the UV-VIS spectrometer, where no significant deviation in absorbance was found. Therefore, it stands to reason that the difference of the absolute values is due to the two different measuring devices/techniques.

This difference could be caused by the different emission sources of the two systems. The UV-VIS spectrometer uses broadband radiation sources that are divided into a narrow spectrum by the use of monochromators, while the laser absorption spectrometer uses narrow-band laser emission. It is remarkable that the determined absorbances show a very high linear correlation over the entire measurement range of $0.18{\;\mathrm{cm}}^{-1}$ to $0.96{\;\mathrm{cm}}^{-1}$.

## 4. Conclusions and Outlook

We present an organic DFB laser-based absorption spectrometer. We showed that the emission based on partial wave decoupling in top and bottom direction can be used for emission monitoring. The decoupling of both partial waves of a second-order DFB resonator can be correlated. The emission ratio for a second-order DFB resonator based on a planar waveguide with symmetry in refractive index was predicted in the literature to be ½, which was confirmed within our experiments.

This correlation can be used to build a DFB laser absorption spectrometer with no need for a beam splitter. The ability to integrate a monitorable laser can be used not only in absorption spectroscopy, but in a wide range of miniaturized measurement systems.

By means of a colorimetric phosphate reaction, an application example was demonstrated. The laser absorption spectrometer was compared to a conventional UV-VIS spectrometer. The application example showed that the organic laser absorption spectrometer yields comparable absorbance values with similar signal to noise ratio as a commercial spectrometer. The possibility of alternative pump sources, such as inorganic laser diodes and light emitting diodes, simplifies the entire experimental setup. Thus, an organic DFB laser absorption spectrometer is ideally suited for optofluidic molecular analysis applications.

**Figure 9:**
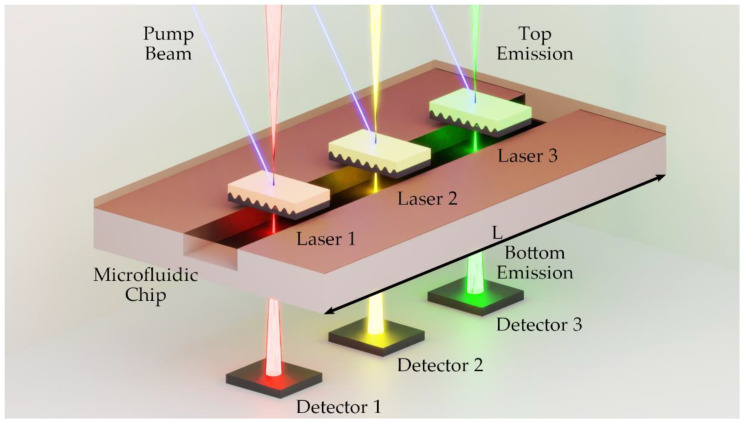
Optofluidic chip version of an organic DFB laser array-based absorption spectrometer.

An absorption spectrometer using a second-order organic DFB laser as a surface emitter is ideally suited for miniaturization (see Figure 9). The sandwich design and the surface emission offer the possibility to integrate a laser source directly onto a microfluidic channel. Our work highlights the integrability by enabling the laser source to be monitored using two-sided emission. Further research can now focus on the integration of the laser source into lab-on-a-chip systems as suggested in Figure 9. It is possible to integrate various organic lasers into an optofluidic chip. By the use of individual emission wavelengths from the entire visible spectrum, it would not only be possible to detect different analytes, but it may also enable the simple implementation of secondary wavelengths for background and offset correction.

## 5. Patents

Karnutsch, C.; Knyrim, J. On-Chip Absorption Sensor for Determining a Concentration of a Specimen in a Sample. U.S. Patent 11,085,865, B2, 7 November 2019.

## Figures and Tables

**Table 1:** Slope, offset and $R^{2}$ of the regression lines from Figure 7b.

Spectrometer Technique	Slope $\left[\frac{l}{g\cdot cm}\right]$	Offset $\left[\frac{1}{m}\right]$	$R^{2}$
laser absorption	528±19.7	16.197±0.484	99.92%
UV-VIS	548±1.19	17.263±0.029	99.73%

## Data Availability

The data presented in this study are available on request from the corresponding author.
